# Reframing child abuse and neglect research through a social work lens: a global bibliometric and thematic evolution analysis (2000–2025)

**DOI:** 10.3389/fped.2026.1829237

**Published:** 2026-07-20

**Authors:** Fatih Cebeci

**Affiliations:** 1 Department of Social Work, School of Health Sciences, Istanbul Medipol University, Istanbul, Türkiye; 2 UNEC Social Work and Social Innovations Research Center, Azerbaijan State University of Economics, Baku, Azerbaijan

**Keywords:** bibliometric analysis, child abuse, child maltreatment, child neglect, child protection, child welfare, science mapping, social work

## Abstract

**Introduction:**

Child neglect and abuse research has expanded rapidly, yet its development has rarely been mapped through a social work lens centered on child welfare and child protection systems. This study provides a bibliometric mapping of the child neglect and abuse literature within the social work context from 2000 to 2025.

**Methods:**

Records were retrieved from the Web of Science Core Collection and Scopus, merged, deduplicated, and screened, yielding a final dataset of 6,132 journal articles and reviews. Using bibliometrix and VOSviewer, annual publication and citation trends, co-authorship networks involving authors, institutions, and countries, co-citation structures, bibliographic coupling, keyword co-occurrence with overlay visualization, and thematic evolution across three periods (2000-2012, 2013-2019, and 2020-2025) were examined.

**Results:**

Publication output showed sustained growth, increasing from 109 publications in 2000 to 362 in 2025, with a compound annual growth rate of approximately 5%. Collaboration was concentrated in high-income countries, with the United States serving as a central hub strongly connected to the United Kingdom, Canada, and Australia. Conceptual mapping indicated a stable core around child abuse and child maltreatment, alongside a shift toward system- and prevention-oriented themes such as child welfare and child protection. Foster care emerged as a prominent motor theme, whereas juvenile justice appeared as a niche theme.

**Conclusion:**

The findings demonstrate that the field has expanded while progressively re-centering on welfare and protection systems. The study provides an empirical baseline for future interdisciplinary research and practice-oriented knowledge translation.

## Introduction

Child neglect and abuse are widely recognized as major threats to children's physical, psychological, and social wellbeing worldwide. The World Health Organization defines child maltreatment as abuse and neglect occurring among children under 18 years of age, including physical and/or emotional ill-treatment, sexual abuse, neglect, negligence, and commercial or other exploitation that results in actual or potential harm to a child's health, survival, development, or dignity (1). Meta-analytic and epidemiological evidence indicates that a substantial proportion of children experience at least one form of maltreatment during childhood, with long-term associations with mental health problems, chronic disease risk, substance use, and later economic disadvantage across the life course (2–4). The impacts of child neglect and abuse extend well beyond early developmental periods and often persist into adolescence and adulthood. Longitudinal research links childhood maltreatment to elevated risks of depression, anxiety, substance use problems, and impairment related to mental and physical health in adulthood (5). This body of evidence supports a shift away from viewing maltreatment solely as an individual or family-level problem and toward prevention-oriented responses embedded in public health, child welfare, and social service systems (1, 3, 6).

Within this research landscape, social work scholarship engages with child neglect and abuse primarily through child welfare service delivery, child protection decision-making, and family support interventions. Consistent with person-environment and structural perspectives, Pelton (2015) emphasizes the continuing role of poverty and material hardship in child maltreatment (7), while Bywaters et al. (2016) show how child welfare intervention rates are patterned by deprivation and identity-related inequalities. From this perspective, maltreatment is better understood as a problem shaped by multilevel social and institutional conditions rather than as an isolated family pathology. These conditions include poverty, social exclusion, structural inequality, identity-related disparities, and service-system constraints (8). Parton (2014) argues that social work and child protection have become closely intertwined within changing political and policy contexts, particularly in England (9). This helps explain why child protection systems, foster care arrangements, family support services, and child welfare policies constitute key institutional arenas through which social work research examines and responds to neglect and abuse. At the same time, Featherstone et al. (2018) call for a more social model of child protection, one that links the protection of children with family support, inequality, social isolation, and humane practice (10).

Recent scholarship also points to a broader conceptual framing in the literature. The field has long used specific labels such as child abuse and child neglect. Contemporary research, however, also makes extensive use of broader and more system-oriented constructs such as child maltreatment, child welfare, and child protection. Dubowitz (2013), for example, argues that neglect should be understood not only through parental omissions but also through whether children's basic needs are adequately met (11). Font and Berger (2015) further show how maltreatment and child protective services involvement are linked to children's developmental trajectories. This conceptual expansion reflects a shift away from narrowly incident-based framings and situates neglect and abuse within the functioning of child welfare systems, preventive social policy, and multidisciplinary service coordination (12).

Parallel to the rapid growth of the field, several bibliometric studies have attempted to map the research landscape of child maltreatment. Tran et al. (2018), for example, examined global publication trends in child maltreatment (13), while Vega-Arce et al. (2019) focused specifically on research performance and trends in child sexual abuse (14). More recent subfield-specific work has mapped childhood emotional abuse research (15), and other studies have provided broader descriptive or science-mapping overviews based on single databases (16, 17). Karadağ and Evinç (2025) also examined general research tendencies in child abuse and neglect and discussed the relevance of bibliometric findings for social work practice (18). However, most existing studies are either broad field-level mappings or subfield-specific analyses. They do not systematically place social work practice, child welfare systems, child protection decision-making, and the profession's person-in-environment orientation at the center of the bibliometric design. Consequently, a comprehensive mapping that prioritizes the social work domain remains a significant gap in the literature.

In addition, prior bibliometric studies in this area have often relied on single-database designs, which may limit coverage and introduce index-specific bias. As Aria and Cuccurullo (2017) note, bibliographic databases do not cover scientific fields and journals in the same way, making database selection an important methodological decision in bibliometric research (19). To address this limitation, this study integrates records from both the Web of Science Core Collection and Scopus. This approach broadens coverage and reduces, although does not eliminate, database-specific coverage bias in mapping the social work-oriented child neglect and abuse literature. It also allows for a more comprehensive examination of thematic continuity, collaboration structures, and intellectual organization within the field. Despite the rapid growth and thematic diversification of child neglect and abuse research, a systematic and multi-layered mapping explicitly situated within the social work context remains limited. Existing bibliometric studies have generated valuable descriptive and science-mapping insights, yet they have given less attention to how core concepts persist, shift, and become re-centered over time within a specifically social work-oriented frame. Methodological work on bibliometric analysis shows that science mapping and temporal analysis are useful for tracing conceptual structures, thematic shifts, and the evolution of research fields (19, 20). Applying this approach to child neglect and abuse research makes it possible to examine how the literature has reorganized around child welfare and child protection frameworks over time.

### The present study

In response to these limitations, this study provides a comprehensive bibliometric mapping of the child neglect and abuse literature within the social work context between 2000 and 2025. The analysis is based on an integrated dataset constructed through the systematic retrieval and deduplication of records from the Web of Science Core Collection and Scopus databases. In this study, the social work context refers to scholarship that explicitly engages with child welfare systems, child protection mechanisms, service delivery structures, and policy relevant intervention frameworks. The study moves beyond descriptive productivity indicators by combining growth analysis with collaboration networks, co-citation structures, bibliographic coupling patterns, keyword mapping, and thematic evolution analysis across three time periods. This multi-layer design enables the identification of thematic continuity, conceptual re framing, and shifts in centrality within the field. The study addresses the following research questions:

RQ1. What patterns of annual publication output are observed between 2000 and 2025, and how are these patterns situated within the development of the field?
RQ2. How are growth-rate indicators and temporal shifts in publication output associated with the expansion and maturation of the literature?
RQ3. How are authors, institutions, and countries positioned in collaboration networks, and how is knowledge production organized in the field?
RQ4. What intellectual and theoretical core emerges from co-citation and bibliographic coupling analyses?
RQ5. What dominant themes and conceptual clusters characterize the literature from a social work perspective?
RQ6. How have these themes evolved over time, and what continuities and turning points emerge across early, middle, and recent periods?

## Methods

### Research design

This study was designed as a descriptive and exploratory bibliometric analysis to examine the development, thematic focus, and structural characteristics of the child neglect and abuse literature within the social work context. Bibliometric methods are well suited to analyzing large bodies of scientific publications, identifying intellectual structures, and tracing the development of research fields over time (20). This data-driven approach was adopted to reduce researcher bias in the review process and to enhance the objectivity of the evaluation (21). Science mapping techniques were used to visualize bibliographic relationships and examine the conceptual and social organization of the field (19, 22).

In line with recent bibliometric protocols, the analysis followed a structured workflow involving data retrieval, data cleaning, preprocessing, network construction, visualization, and interpretation (23). Performance analysis was combined with co-authorship, co-citation, bibliographic coupling, keyword co-occurrence, and thematic mapping procedures. This combination is consistent with bibliometric approaches that use publication trends, citation patterns, collaboration structures, and thematic indicators to understand the development of a research field (24). This design made it possible to examine publication growth, influential contributors, collaboration patterns, intellectual linkages, and the thematic development of child welfare scholarship within the social work-oriented child neglect and abuse literature.

### Data sources

The bibliometric dataset was compiled from two major international indexing platforms that provide broad coverage and standardized bibliographic records in the social sciences and child protection domains: the Web of Science Core Collection and Scopus. These sources were preferred because they extensively index social work research together with interdisciplinary scholarship addressing child neglect, child abuse, and child welfare systems. Using both databases in combination was intended to maximize retrieval breadth and to minimize database-specific coverage bias, given that each index offers overlapping yet complementary journal inclusion. All database searches were performed on 10 December 2025. Retrieved records were exported in BibTeX format to facilitate accurate data cleaning, integration, and subsequent bibliometric processing. Because database indexing is continuously updated, some publications released toward the end of 2025 may not have been fully indexed at the time of data extraction. Consequently, studies published after the retrieval date or not yet indexed by 10 December 2025 were not captured in the final dataset.

### Search strategy

#### Web of science

In the Web of Science Core Collection, the search was conducted in the Topic field, which includes title, abstract, author keywords, and Keywords Plus. The search strategy was constructed to capture core child neglect and abuse concepts together with terms representing social work and child welfare systems. The search query was structured as follows:

TS = [(“child abuse” OR “child neglect” OR “child maltreatment” OR “child sexual abuse” OR “child physical abuse” OR “child emotional abuse”) AND (“social work” OR “child welfare” OR “child protection” OR “protective services” OR “foster care”)]

Results were limited to publication years 2000–2025, document types of articles and review articles, and English language. Index coverage was restricted to the Social Sciences Citation Index and Science Citation Index Expanded to ensure high quality bibliographic records. Conference proceedings, proceeding papers, data papers, book chapters, and retracted publications were excluded.

#### Scopus

In Scopus, the search was conducted in the Title Abstract Keywords field to ensure conceptual consistency with the Web of Science strategy. The search query was:

TITLE ABS KEY [(“child abuse” OR “child neglect” OR “child maltreatment” OR “child sexual abuse” OR “child physical abuse” OR “child emotional abuse”) AND (“social work” OR “child welfare” OR “child protection” OR “protective services” OR “foster care”)]

Filters were applied for publication years 2000–2025, document type article or review, source type journal, and English language.

### Study selection and screening process

**Figure 1:**
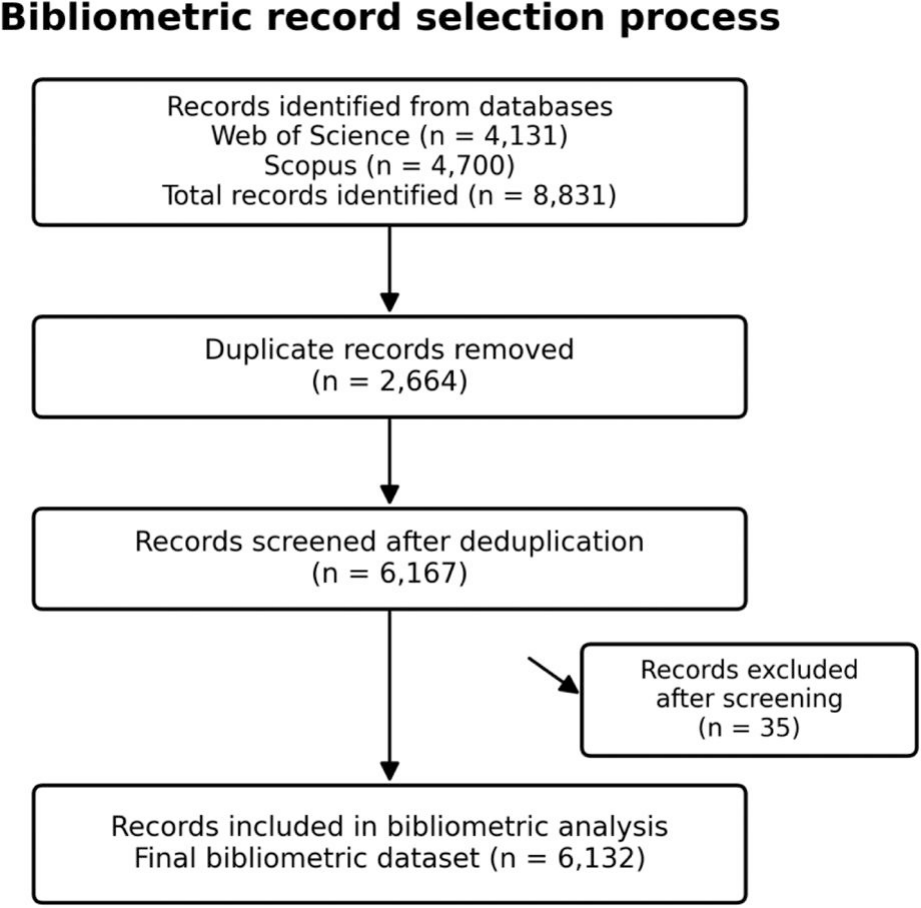
Flow diagram of bibliometric record selection and final dataset construction. The diagram summarizes the database search, duplicate removal, screening, exclusion, and final inclusion of bibliographic records used in the bibliometric analysis.

Records retrieved from both databases were evaluated according to predefined inclusion and exclusion criteria. Studies were included if they were published between 2000 and 2025, were peer reviewed journal articles or review articles, were written in English, and explicitly addressed child neglect and or abuse within social work, child welfare, or child protection contexts. Conference papers, book chapters, editorials, and studies focused primarily on adult populations were excluded. To ensure topical relevance, titles and abstracts were screened for alignment with child focused social work and protection contexts. The bibliometric dataset used in this study was obtained through systematic searches of the Web of Science Core Collection and Scopus databases. The initial search identified 4,131 records from Web of Science and 4,700 records from Scopus, yielding a total of 8,831 bibliographic records. After merging the two datasets, duplicate records were removed using DOI-based and title-based deduplication procedures. This process resulted in the removal of 2,664 duplicate records. The remaining 6,167 records were screened according to the predefined inclusion and exclusion criteria. During this screening process, 35 records were excluded because they did not meet the eligibility criteria for the final bibliometric dataset. The final dataset therefore comprised 6,132 publications, which were included in the bibliometric analyses. The identification, deduplication, screening, and inclusion process of bibliographic records is presented in Figure 1.

### Data integration and deduplication

Bibliographic records from Web of Science and Scopus were imported into the R environment and processed using the bibliometrix package (19). Duplicate records were identified through a two-stage procedure. In the first stage, automated matching was performed using the Digital Object Identifier field. For records with missing or incomplete DOI information, titles were converted to lowercase, stripped of punctuation, and normalized for exact title matching in the second stage. A random subset of records was manually reviewed to verify deduplication accuracy. After duplicate removal and eligibility screening, the final dataset of 6,132 publications was used for all subsequent analyses.

### Bibliometric analysis procedures

A comprehensive bibliometric framework integrating performance analysis and science mapping techniques was employed to examine the structural and conceptual development of the child neglect and abuse literature within the social work context. Descriptive and temporal analyses were conducted in the R environment using the bibliometrix package. Annual publication output was calculated, publication trends were examined, and the compound annual growth rate (CAGR) was computed to evaluate long-term growth dynamics. Productivity and citation-based indicators were used to identify the most productive and visible authors, institutions, countries, and journals. To map the intellectual and collaborative structure of the field, science mapping techniques were applied (25). Co-authorship, co-citation, bibliographic coupling, and keyword co-occurrence networks were constructed. Network visualizations were generated using VOSviewer version 1.6.20 (22). Different threshold values were applied depending on the type and unit of bibliometric analysis in order to ensure reproducibility and preserve network interpretability. The author keyword co-occurrence analysis was conducted using author keywords, the full counting method, and a minimum threshold of five occurrences. Source co-citation analysis was performed with a minimum threshold of 20 citations per cited source, while author co-citation analysis used a minimum threshold of 10 citations per cited author. For author-level bibliographic coupling, a minimum threshold of 10 shared references was applied. Co-authorship networks at the author, institutional, and country levels were generated using the full counting method. The minimum document threshold was set at 6 for institutional co-authorship and 5 for country co-authorship. Thresholds were selected to retain substantively interpretable network structures and to prevent excessive fragmentation. These procedures were used to construct co-authorship, co-citation, bibliographic coupling, and keyword co-occurrence networks across the relevant units of analysis.

For the keyword co-occurrence analysis, author keywords were used as the primary analytical unit, and the full counting method was applied. To enhance conceptual consistency and reduce terminological fragmentation, a thesaurus file was developed prior to mapping to harmonize synonyms, abbreviations, and spelling variants. This preprocessing step was consistent with bibliometric data-cleaning procedures used to improve the quality and interpretability of co-word and co-occurrence analyses (19, 22). For transparency, the thesaurus file harmonized spelling variants, abbreviations, and closely equivalent keyword labels. For example, “child maltreatment,” “child mal-treatment,” and “maltreatment of children” were merged under “child maltreatment”; “child protection services” and “protective services” were merged under “child protection services”; “out-of-home care” and “out of home care” were merged under “out-of-home care”; “posttraumatic stress disorder,” “post-traumatic stress disorder,” and “PTSD” were merged under “post-traumatic stress disorder”; and “COVID 19” and “COVID-19” were merged under “COVID-19.” The minimum occurrence threshold for keyword inclusion was set to five in order to filter out infrequent terms and retain more recurrent and interpretable themes in the keyword network. In the network visualizations, node size represents the relative weight of each unit, while link strength indicates the intensity of relationships between nodes, following the visualization logic of the VOS mapping technique (22). Temporal dynamics were examined through overlay visualization based on average publication year, which enabled the chronological positioning of topics within the keyword network. Association strength normalization and the default VOSviewer clustering algorithm were applied to obtain a normalized and interpretable representation of the research landscape (22). These procedures are consistent with science mapping approaches that use bibliographic relationships, co-occurrence patterns, and visualization techniques to examine the conceptual structure and development of research fields (21, 22, 26).

## Results

### Quantitative growth dynamics

#### Annual publication and citation trends, 2000–2025

The temporal distribution of publications on child neglect and abuse within the social work context shows a steady upward pattern between 2000 and 2025. Annual publication counts increase gradually in the early years and display a more pronounced rise after 2008.

**Figure 2:**
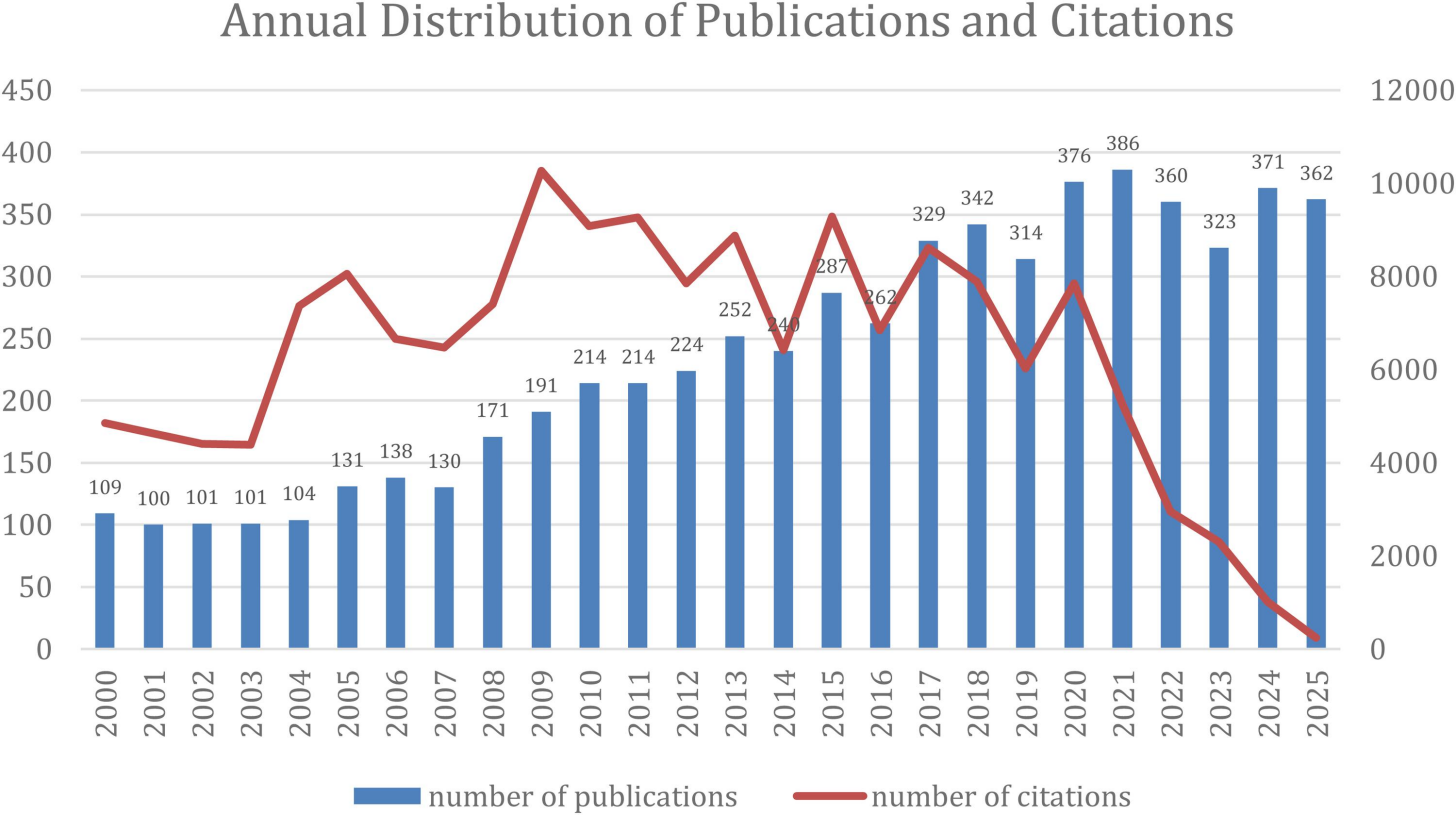
Annual publication output and annual citation counts in the child neglect and abuse literature within the social work context, 2000–2025. Bars indicate the number of publications indexed in each publication year. The citation series represents the total number of citations received in each calendar year by the publications included in the dataset up to that year. Therefore, lower citation counts in recent years should be interpreted in light of citation time-lag effects. The 2025 publication count should also be interpreted cautiously because late-2025 publications may not yet have been fully indexed at the time of retrieval.

As shown in Figure 2, publication output followed an overall upward trajectory across the study period. After relatively modest and fluctuating publication levels in the early 2000s, the increase became more visible from approximately 2008 onward and gained further momentum after 2017. By 2025, the annual number of publications reached 362, indicating substantial growth in research activity within the field. The trajectory of annual citation counts followed a different temporal pattern. Annual citations rose markedly between 2008 and 2012 and then fluctuated in the following years. This citation line should not be interpreted as a cumulative citation curve; rather, it represents the total number of citations received in each calendar year by the publications included in the dataset. Therefore, fluctuations in the citation series reflect annual citation activity rather than a continuously accumulating citation total. Thus, Figure 2 shows the coexistence of sustained publication growth and uneven annual citation activity across the study period.

### Growth rate indicators: CAGR and temporal shifts

To quantify the increase in publication output, the compound annual growth rate was calculated for the period from 2000 to 2025. The calculation was based on the number of publications in 2000 (*n* = 109) and in 2025 (*n* = 362). The results indicated an overall compound annual growth rate of approximately 5% across the 25-year period. Because the 2025 publication count may have been affected by incomplete late-year indexing, a sensitivity calculation was also performed using 2024 as the endpoint. Using 109 publications in 2000 and 371 publications in 2024, the CAGR was approximately 5.2%, which was consistent with the main estimate and supported the robustness of the overall growth pattern. During the early 2000s, publication output remained relatively modest and fluctuated at lower levels. From approximately 2008 onward, a clearer upward pattern became visible. This growth became more regular during the 2010–2015 period and increased more sharply after 2016, with annual output reaching its highest levels after 2020. These temporal patterns describe the changing pace of publication activity across the study window. To further examine how quantitative expansion relates to scientific influence within the field, the next section analyses citation performance and the most highly cited publications.

### Most influential publications and citation distribution

Table 1 presents the ten most highly cited publications in the child neglect and abuse literature within the social work context. The publication years of these studies are concentrated primarily between 2004 and 2020, with a visible clustering in the 2009–2016 period.

**Table 1:** Top ten most cited publications in the child neglect and abuse literature within the social work context, 2000–2025.

Rank	Authors	Year	Journal	Total citation
1	Brown et al.	2020	*Child Abuse and Neglect*	997
2	Burns et al.	2004	*Journal of the American Academy of Child and Adolescent Psychiatry*	951
3	Stith et al.	2009	*Aggression and Violent Behavior*	862
4	Fang et al.	2012	*Child Abuse and Neglect*	709
5	Sullivan and Knutson	2000	*Child Abuse and Neglect*	694
6	Chaffin et al.	2004	*Journal of Consulting and Clinical Psychology*	614
7	Coulton et al.	2007	*Child Abuse and Neglect*	579
8	Kim et al.	2017	*American Journal of Public Health*	454
9	Fonagy et al.	2007	*Journal of Child Psychology and Psychiatry and Allied Disciplines*	438
10	Herrenkohl et al.	2008	*Trauma, Violence, and Abuse*	430

Publications are ranked by total citation counts as of 10 December 2025, the date on which records were retrieved from Web of Science Core Collection and Scopus.

As shown in Table 1, the most highly cited studies were published in high-impact journals across multiple disciplines. These publications address a broad range of issues related to child maltreatment, including intervention, mental health, risk factors, family and environmental conditions, and child welfare system involvement. The citation distribution within the combined dataset is highly skewed. While a large share of publications received relatively few citations, a small subset of studies accumulated markedly higher citation counts. The top ten publications account for a disproportionate share of total citations in the field. When examined by publication year, earlier and mid-period studies generally tend to display higher cumulative citation counts than more recent publications. This pattern is consistent with the time-dependent nature of citation accumulation. Following the citation-based findings, the next section examines the collaboration structures underlying scientific production at the author, institutional, and country levels.

### Collaboration structures and the organization of scientific production

Co-authorship analyses indicate that research on child neglect and abuse within the social work context is produced through collaborative networks that extend beyond individual authors to institutional and international partnerships. These findings describe the structural organization of scholarly production and the scope of collaboration patterns in the field.

#### Author co-authorship network

To examine the structural properties of academic collaboration, an author level co-authorship analysis was conducted for the child neglect and abuse literature within the social work context. The analysis maps patterns of joint authorship and the configuration of collaborative research groups.

**Figure 3:**
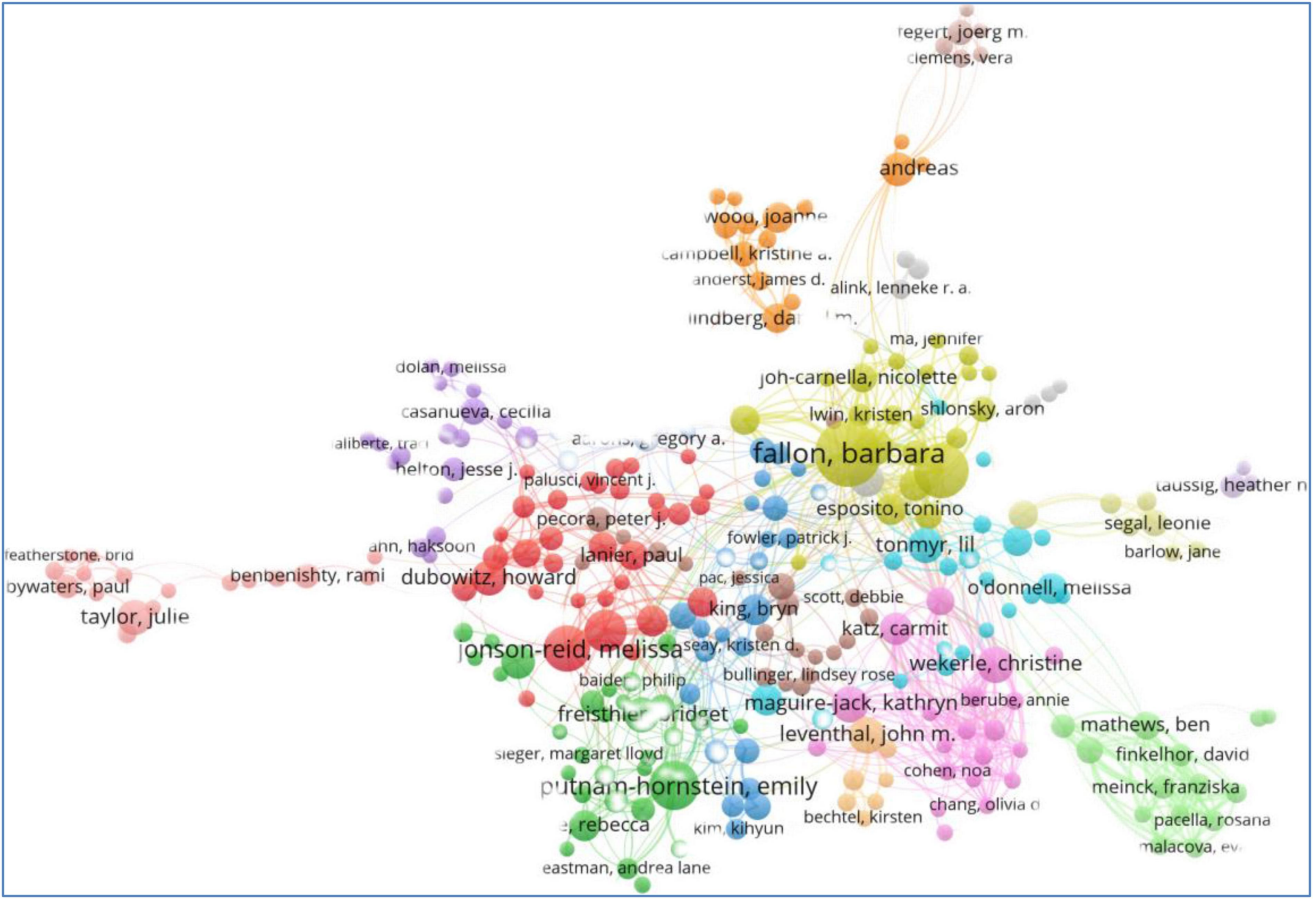
Author co-authorship network in the child neglect and abuse literature within the social work context. Nodes represent authors, node size reflects publication volume, links indicate co-authorship relationships, and colours denote collaboration clusters. The network includes authors with a minimum of five publications and was generated using the full counting method.

The co-authorship map in Figure 3 displays a clustered collaborative structure. Several authors occupy visually prominent positions in the network, including Barbara Fallon, Melissa Jonson-Reid, Howard Dubowitz, Emily Putnam-Hornstein, Kathryn Maguire-Jack, Christine Wekerle, Tonino Esposito, Lil Tonmyr, and Rami Benbenishty. Other visible author groups, including those associated with Paul Bywaters and Brid Featherstone, appear in more peripheral areas of the network. This pattern suggests that research production in the field is not organized around a single author group, but rather around multiple collaborative communities with different institutional and thematic connections.

#### Institutional co-authorship network

To complement the author level findings, an institutional co-authorship analysis was conducted to examine the organizational structure of scientific production in the child neglect and abuse literature within the social work context. This analysis identifies the universities and research institutions around which publication activity is concentrated.

**Figure 4:**
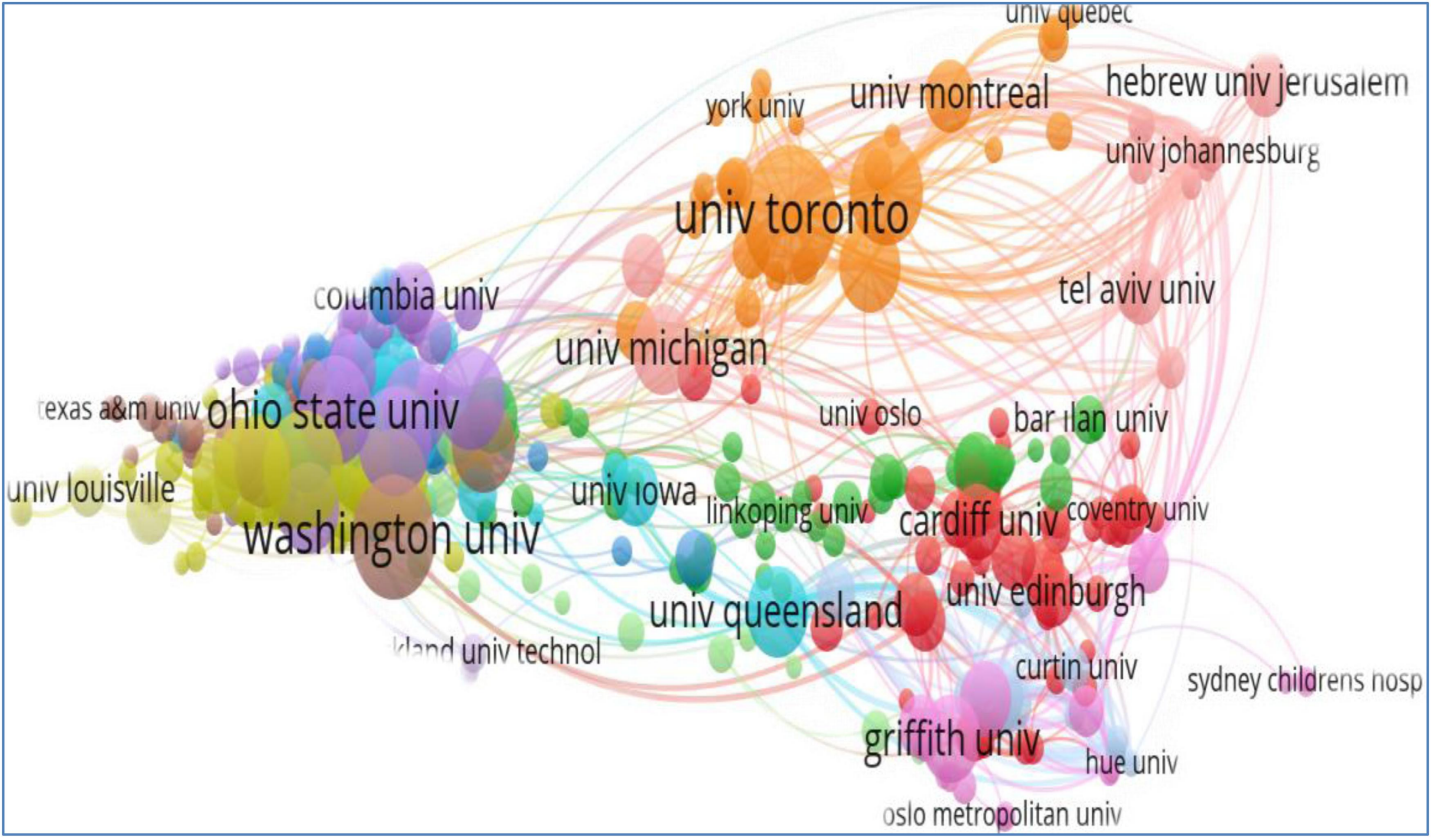
Institutional co-authorship network in the child neglect and abuse literature within the social work context.

The institutional network displayed in Figure 4 reveals a clustered pattern of collaboration among universities. Several institutions occupy visually prominent positions in the network, including the University of Toronto, the University of Washington, Ohio State University, and the University of Michigan. These institutions show extensive collaborative linkages with multiple partner organizations. Additional universities such as Cardiff University, the University of Edinburgh, the University of Queensland, and Griffith University appear as well-connected nodes within the broader collaboration structure. In other areas of the network, smaller institutional clusters are observable, indicating more localized patterns of collaboration. This pattern suggests that institutional collaboration in the field is organized around a limited number of highly connected university hubs, alongside smaller regional or topic-specific collaboration groups.

#### International co-authorship network

To extend the institutional level findings, an international co-authorship analysis was conducted to examine collaboration patterns at the country level in the child neglect and abuse literature within the social work context. The analysis maps the extent to which scientific production is conducted through cross national partnerships.

**Figure 5:**
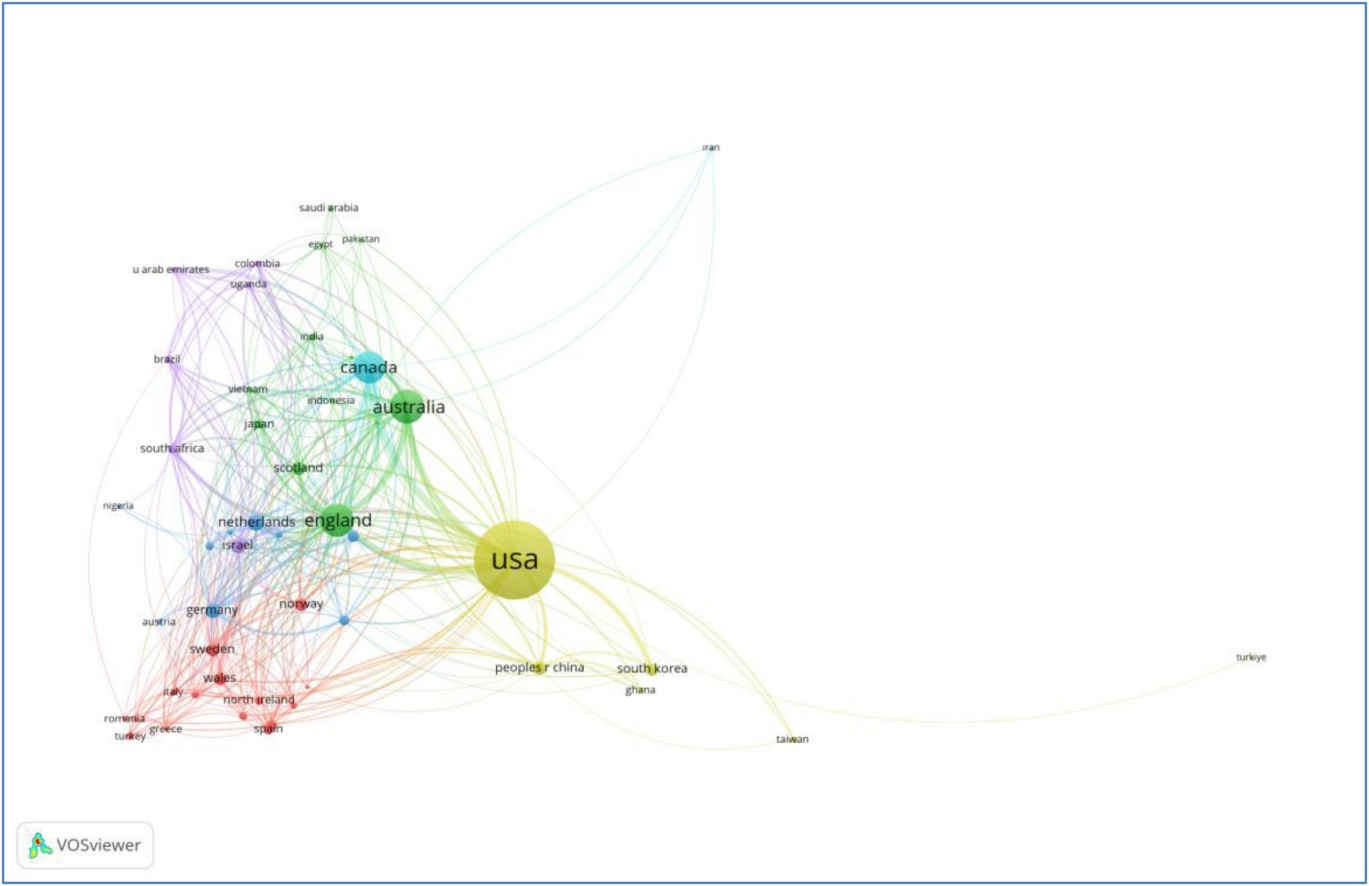
International co-authorship network in the child neglect and abuse literature within the social work context.

The country level network presented in Figure 5 shows a highly interconnected collaboration structure. The United States occupies a visually dominant position in the network in terms of publication volume and link strength. Strong co-authorship ties are visible between the United States and the United Kingdom, Canada, and Australia. Several European countries, including the Netherlands, Germany, Sweden, and Norway, also appear as well-connected nodes linked to multiple international partners. In contrast, countries from Asia, Africa, and South America are represented by smaller nodes and fewer collaboration links within the network. This distribution suggests that international collaboration in the indexed literature is concentrated around a small group of highly connected countries, while many regions remain more peripheral in the collaboration structure.

### Intellectual structure: citation-based networks

To further examine the knowledge base of the field, citation-based analyses were conducted following the assessment of quantitative growth and collaboration patterns. This stage focuses on the distribution of influential references and the structural configuration of the literature using co-citation and bibliographic coupling techniques.

#### Author co-citation network

To identify the intellectual core of the field, an author co-citation analysis was performed based on frequently co-cited authors within the child neglect and abuse literature in the social work context.

**Figure 6:**
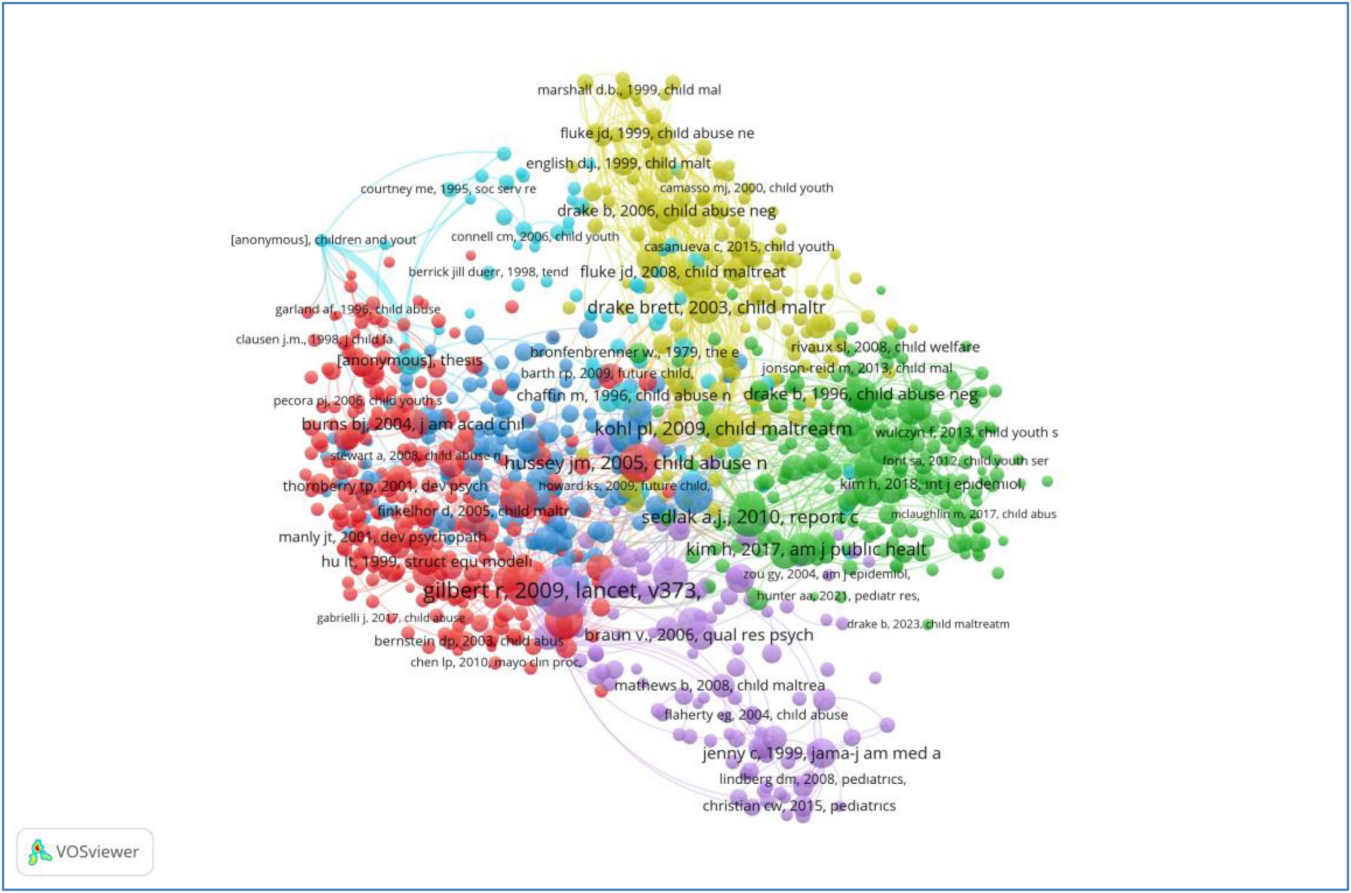
Author co-citation network in the child neglect and abuse literature within the social work context.

The co-citation map presented in Figure 6 displays a structured and clustered intellectual landscape. Authors such as Gilbert, Drake, and Hussey occupy visually prominent positions in the network based on co-citation frequency and link strength. The clustering pattern indicates the presence of multiple thematic groupings within the literature. Some clusters are positioned around clinical and individual level research traditions, whereas others are associated with system level, policy oriented, and public health focused scholarship. These clusters remain interconnected through shared citation linkages across the network. This structure suggests that the intellectual base of the field is shaped by both individual and clinical research traditions and broader system, policy, and public health perspectives.

#### Source co-citation network

To examine the structure of journal level knowledge foundations in the child neglect and abuse literature within the social work context, a source co-citation analysis was conducted.

**Figure 7:**
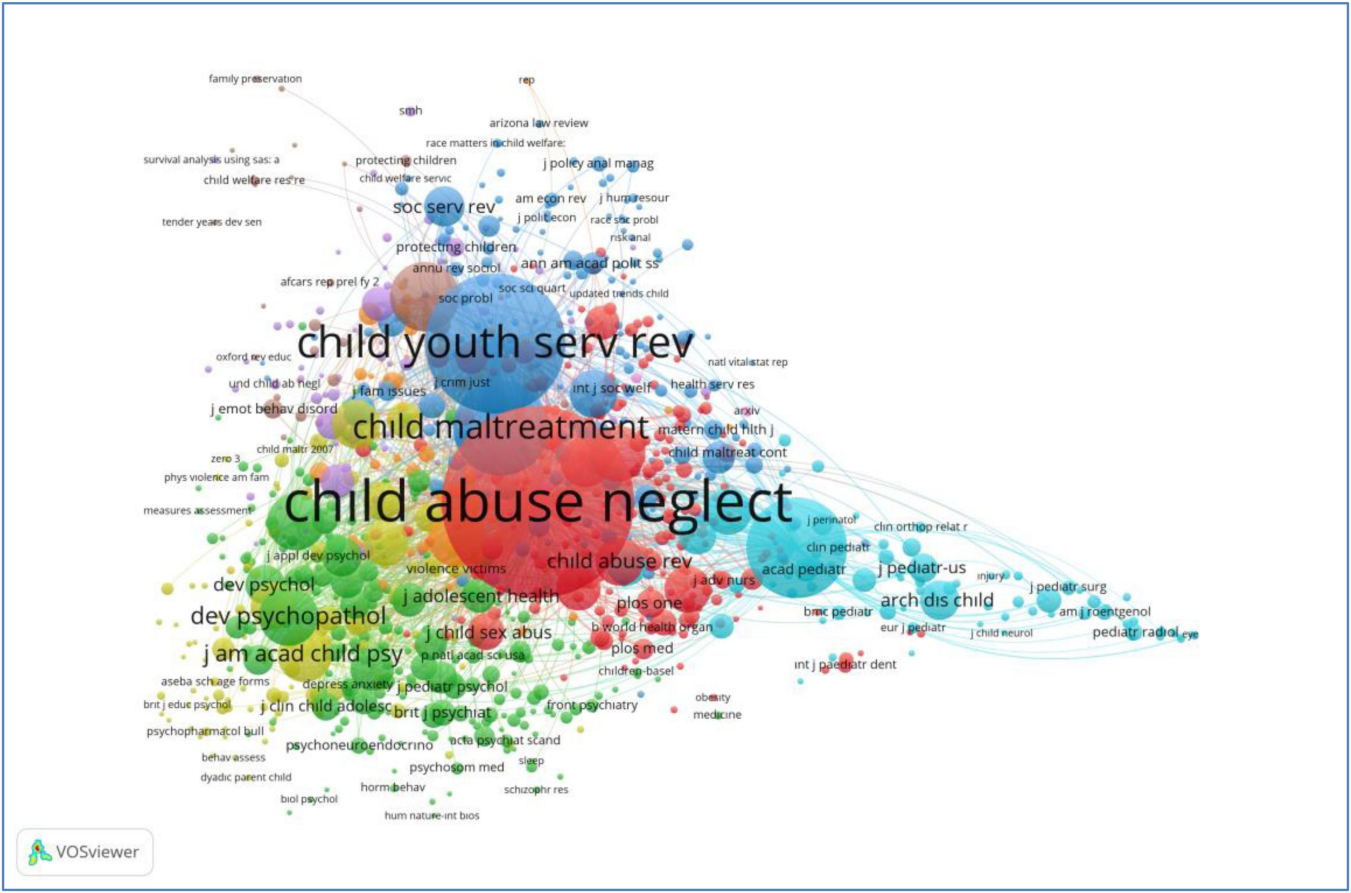
Source co-citation network in the child neglect and abuse literature within the social work context.

The source co-citation map presented in Figure 7 shows a clustered journal landscape. Several journals occupy visually prominent positions in the network, including *Child Abuse and Neglect*, *Child and Youth Services Review*, and *Child Maltreatment*. These sources display strong co-citation linkages within the network. Additional clusters include journals associated with developmental psychology, psychopathology, pediatrics, and public health. The network visualization indicates that journals from multiple disciplinary areas are connected through shared citation patterns. This pattern suggests that the field has a recognizable journal-level core around child maltreatment and child welfare outlets, while also drawing on closely related developmental, clinical, pediatric, and public health literatures.

#### Author bibliographic coupling network

To complement the co-citation findings, an author-level bibliographic coupling analysis was conducted to examine how authors are connected through shared reference bases in the child neglect and abuse literature within the social work context.

**Figure 8:**
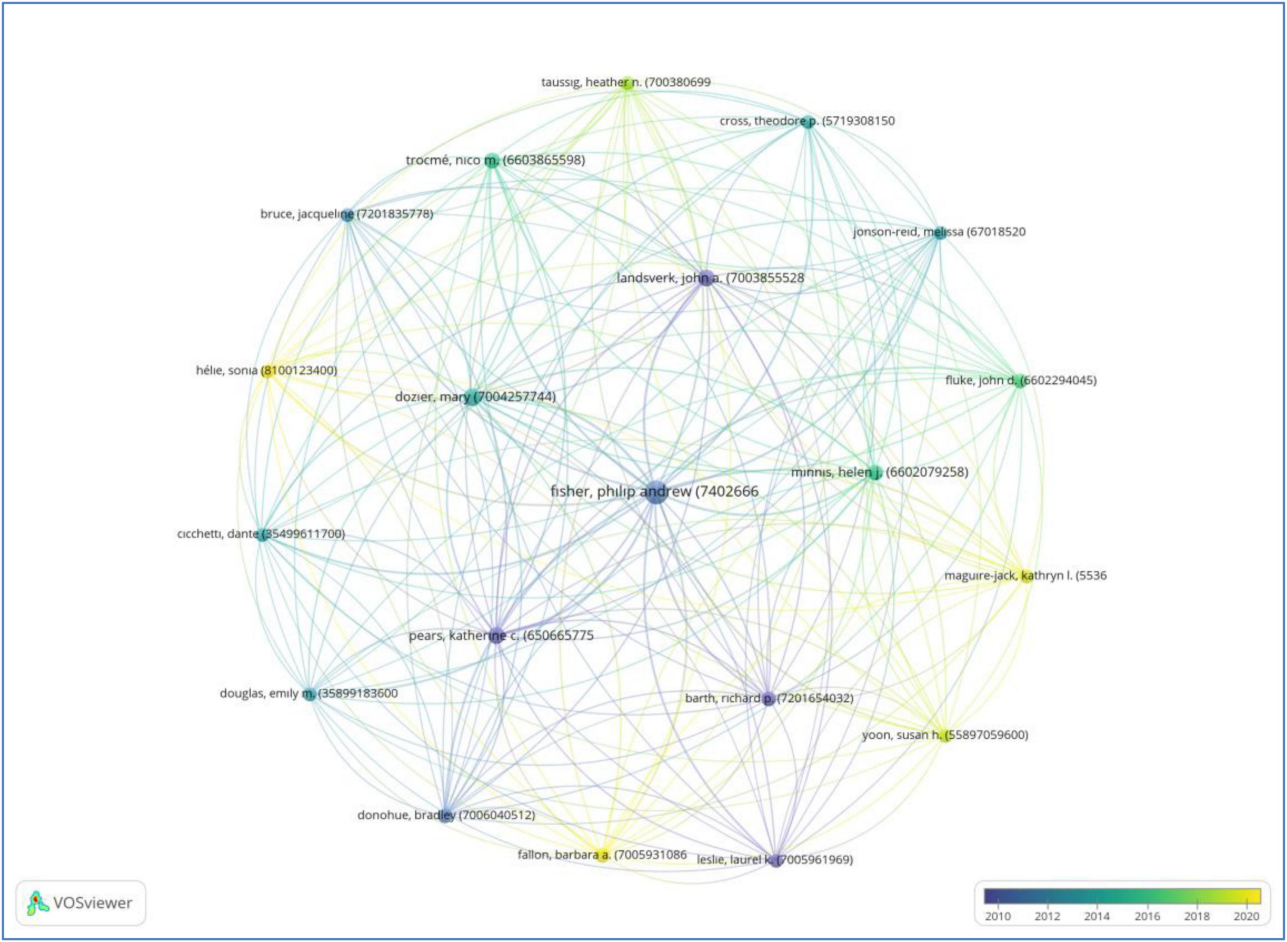
Author-level bibliographic coupling network in the child neglect and abuse literature within the social work context. Nodes represent authors, links indicate bibliographic coupling relationships based on shared references, and node size reflects the relative weight of authors in the network. The colour gradient indicates the average publication year associated with each author's publications.

The author-level bibliographic coupling map presented in Figure 8 shows a dense network of authors connected through overlapping reference lists. Several authors occupy visually prominent or bridging positions in the network, including Philip Andrew Fisher, John A. Landsverk, Katherine C. Pears, Laurel K. Leslie, Richard P. Barth, Melissa Jonson-Reid, John D. Fluke, Kathryn L. Maguire-Jack, and Nico M. Trocmé. The network also includes authors such as Barbara A. Fallon, Heather N. Taussig, Dante Cicchetti, Mary Dozier, and Emily M. Douglas, indicating shared bibliographic foundations across multiple research streams. The temporal colour gradient suggests that earlier bibliographic connections are more visible around authors such as Landsverk, Pears, Leslie, and Barth, whereas more recent linkages appear around authors such as Maguire-Jack, Yoon, Fallon, Hélie, and Taussig. This pattern complements the co-citation findings by showing that the intellectual structure of the field is shaped not only by older co-cited foundations, but also by more recent author communities drawing on overlapping bodies of literature.

### Conceptual structure: keyword based thematic patterns

The conceptual structure and dominant research themes of the literature were examined using a co-occurrence analysis based on author keywords. Author keywords provide direct insight into the conceptual framing of studies and allow the identification of thematic groupings and relationships among key terms. Co-occurrence patterns were analyzed, and temporal variation in keyword usage was further explored through overlay visualization.

#### Author keyword co-occurrence network

To identify frequently co-appearing concepts and thematic clusters, a co-occurrence analysis based on author keywords was conducted for the child neglect and abuse literature within the social work context.

**Figure 9:**
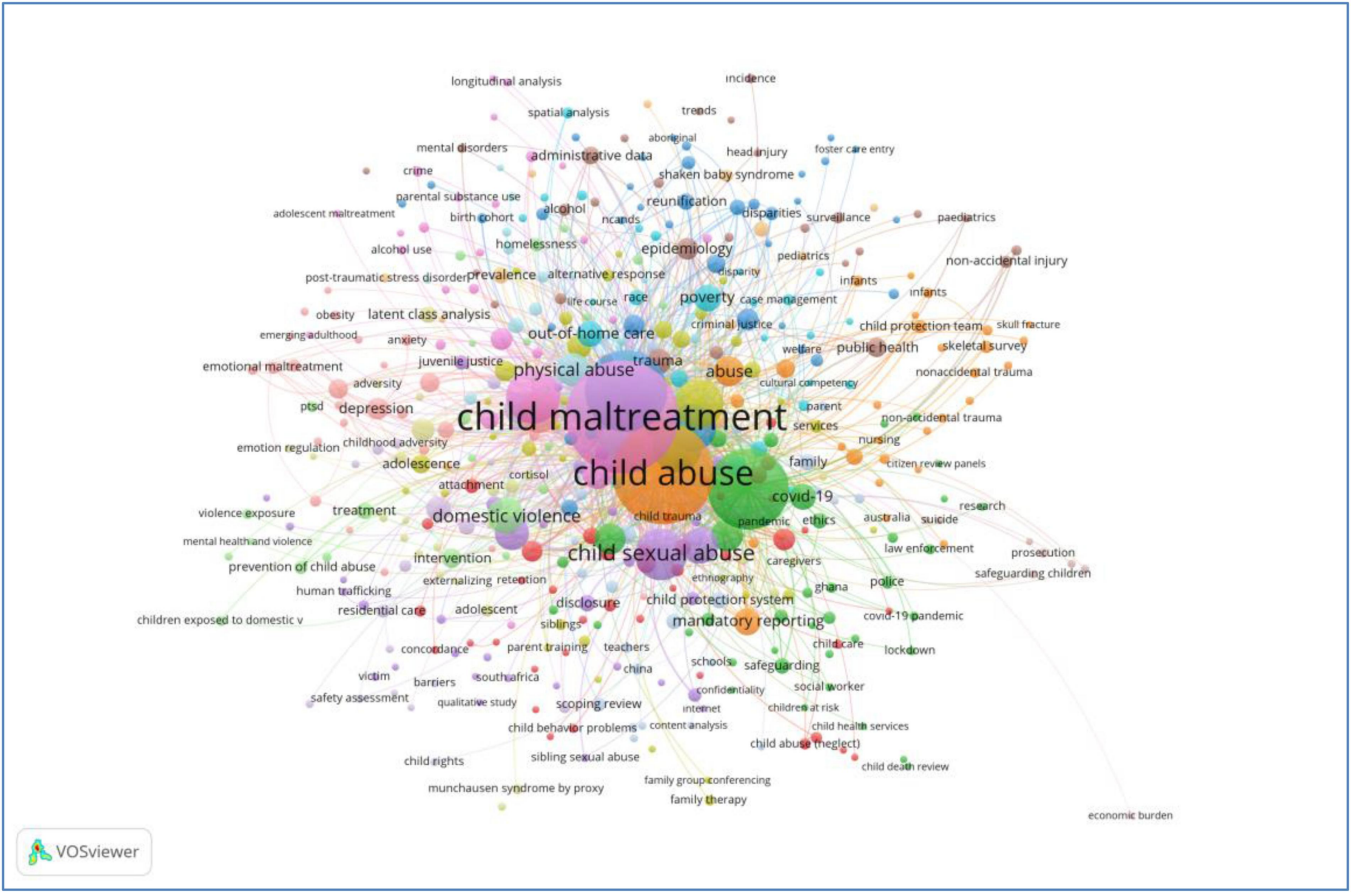
Author keyword co-occurrence network in the child neglect and abuse literature within the social work context.

The network presented in Figure 9 shows a dense conceptual structure centered on several high frequency keywords. Terms such as *child maltreatment*, *child abuse*, *child sexual abuse*, and *physical abuse* occupy central positions in the map, reflecting their high occurrence and extensive linkages with other concepts. These central terms are strongly connected with keywords related to trauma, domestic violence, mental health, intervention, and child protection. Additional thematic groupings are visible around public health-oriented terms such as epidemiology and public health, system related concepts including mandatory reporting, criminal justice, and law enforcement, psychosocial outcomes such as depression, post-traumatic stress disorder, and emotional maltreatment, and care system related terms including out of home care, foster care, and reunification. Within the network, some keywords function as bridging nodes linking multiple thematic clusters, whereas others appear in more peripheral positions with fewer co-occurrence links. The keyword pattern indicates that the field is conceptually organized not only around forms of abuse and maltreatment, but also around outcomes, service responses, legal mechanisms, and child welfare system processes.

#### Overlay analysis: temporal dynamics of thematic emphasis

**Figure 10:**
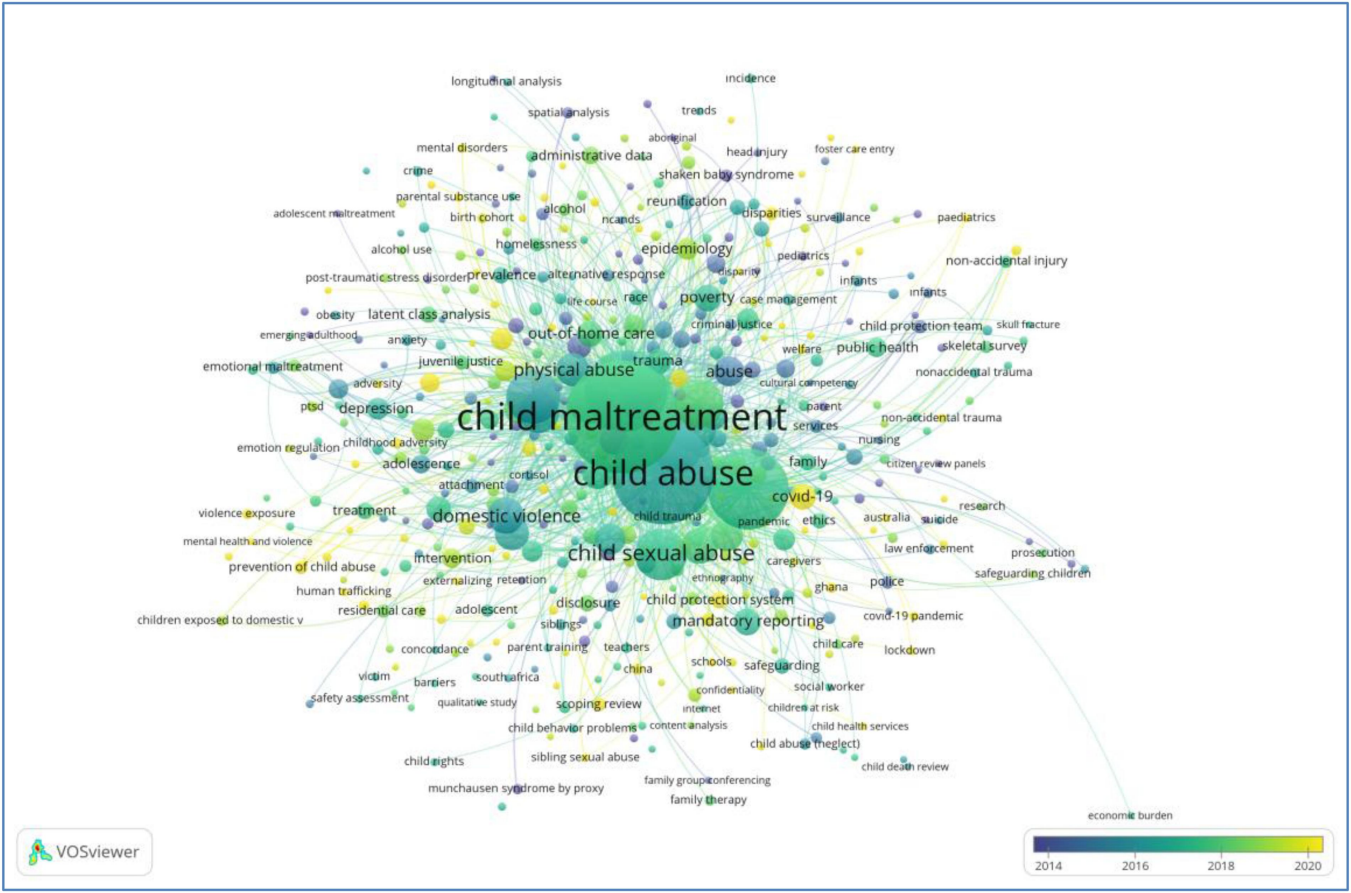
Overlay visualization of the author keyword co-occurrence network in the child neglect and abuse literature within the social work context.

To examine temporal patterns in keyword usage and to capture the time-based dimension of thematic variation, an overlay visualization based on the keyword co-occurrence network was generated (Figure 10).

The overlay map in Figure 10 indicates temporal variation in the prominence of specific keywords. Core terms such as *child maltreatment*, *child abuse*, and *child sexual abuse* remain centrally positioned across the time spectrum, reflecting their sustained presence in the literature.

Earlier appearing keywords are more frequently associated with psychosocial outcome related terms such as *physical abuse*, *domestic violence*, *depression*, *post-traumatic stress disorder*, and *attachment*. In subsequent periods, greater visibility is observed for system and protection-oriented terms including *out of home care*, *mandatory reporting*, *child protection system*, and *intervention*. More recent keywords appear in peripheral areas of the network and include terms such as *COVID-19*, *pandemic*, *public health*, *lockdown*, and *economic burden*. The colour gradient in the overlay visualization reflects the relative average publication year associated with each keyword. This temporal pattern indicates that the literature has gradually expanded from psychosocial outcome-focused topics toward system, protection, and broader public health concerns. Following the keyword-based findings, the next stage of the analysis examines thematic evolution across time periods.

### Thematic evolution analysis

To assess whether the observed increase in publication output corresponds to shifts in thematic emphasis within the child neglect and abuse literature in the social work context, a thematic evolution analysis was conducted. This analysis traces the continuity, repositioning, and relative prominence of major keyword clusters across time periods.

#### General structure of thematic mapping

**Figure 11:**
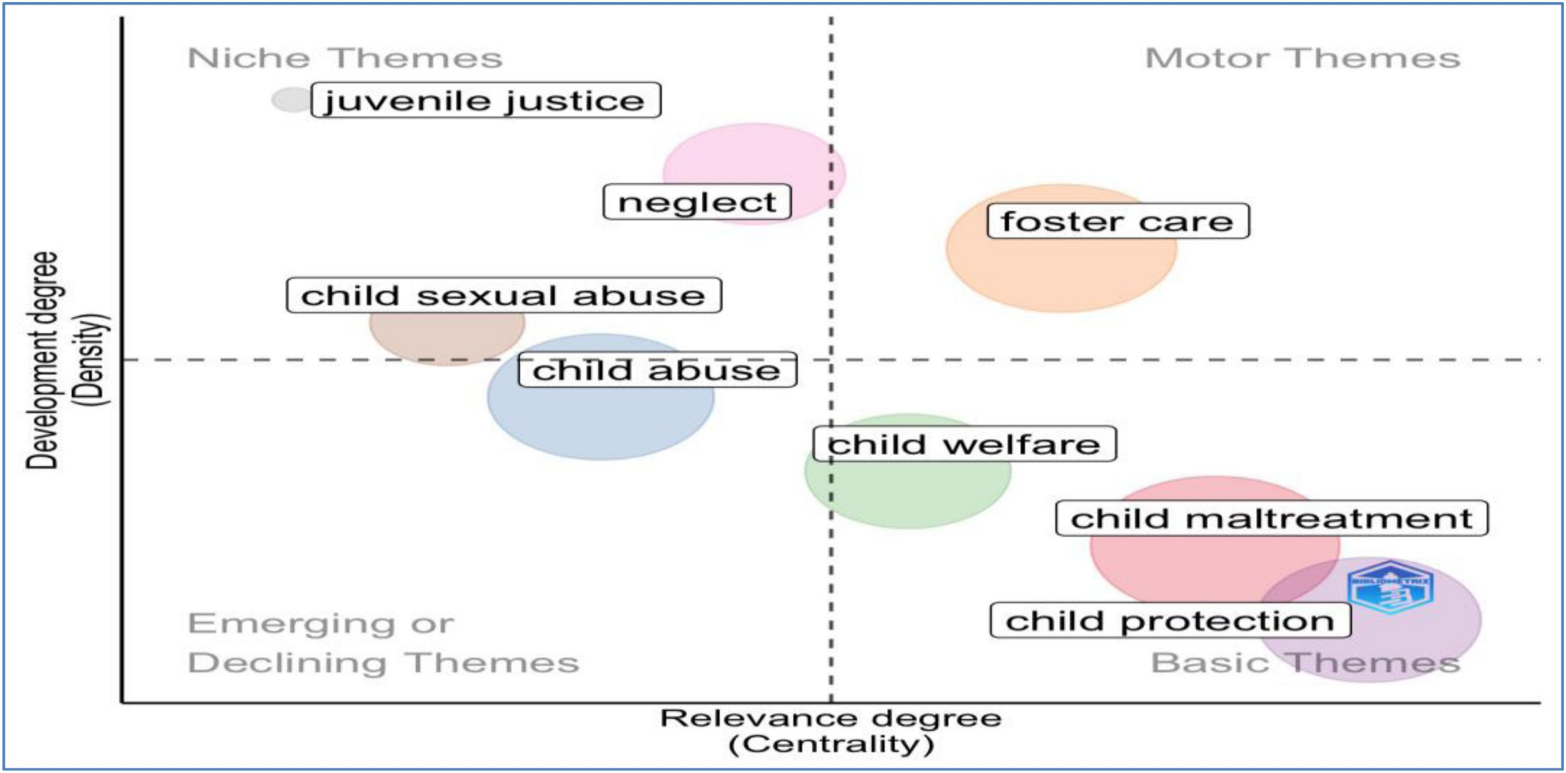
Thematic map of the child neglect and abuse literature within the social work context. The thematic map positions keyword clusters according to centrality and density values. Bubble size represents the relative volume of each theme and colours indicate cluster membership. The upper right quadrant corresponds to motor themes, the lower right to basic themes, the upper left to niche themes, and the lower left to emerging or declining themes.

The thematic evolution analysis was conducted across three predefined temporal slices: 2000–2012, 2013–2019, and 2020–2025. These cut points were data-informed but investigator-defined rather than automatically generated breakpoints. Specifically, the periods were selected after inspecting annual publication trends, changes in keyword centrality and density, and transitions among major keyword clusters across the study window. The first period captures the earlier consolidation of child abuse and maltreatment scholarship, the second reflects the expansion of system- and welfare-oriented terminology, and the third captures the most recent phase marked by stronger visibility of child protection, public health, and post-2020 themes. The resulting thematic structure is presented in Figure 11.

The thematic map in Figure 11 displays several distinct clusters. The motor themes quadrant includes *foster care* as a prominent cluster. The basic themes quadrant contains clusters labelled *child maltreatment*, *child protection*, and *child welfare*. The niche themes quadrant includes *juvenile justice*, while *neglect* appears near the upper central region of the map. Themes such as *child sexual abuse* and *child abuse* are positioned toward the left side of the map, indicating comparatively lower centrality within the network. Differences in density values are also visible across these clusters. The placement of foster care as a motor theme and child welfare, child protection, and child maltreatment as basic themes indicates that the field is organized around both specialized care-related research areas and broader foundational system-level concepts.

#### Quantitative analysis of thematic flows across periods

Within the thematic evolution framework, the weighted inclusion index (Inc_Weighted) was calculated to quantify how themes were transferred and reconfigured across successive periods in the child neglect and abuse literature within the social work context. This indicator reflects the extent to which a theme in one period is retained, expanded, or reduced in the subsequent period. Based on Inc_Weighted values, the five strongest thematic flows between consecutive periods were identified and are presented in Table 2.

**Table 2:** Top five thematic flows in the child neglect and abuse literature within the social work context, 2000–2025.

Rank	Previous theme (CL1)	Subsequent theme (CL2)	Inc_Weighted	Stability
1	Child abuse (2000–2012)	Child abuse (2013–2019)	0.725	0.008
2	Child maltreatment (2013–2019)	Child maltreatment (2020–2025)	0.696	0.008
3	Child welfare (2013–2019)	Child maltreatment (2020–2025)	0.574	0.008
4	Child abuse (2013–2019)	Child protection (2020–2025)	0.558	0.008
5	Child maltreatment (2000–2012)	Intimate partner violence (2013–2019)	0.520	0.012

Inc_Weighted represents the weighted inclusion index, indicating the degree of thematic carryover between consecutive periods. It is the primary indicator used to interpret the strength of thematic flows. Stability reflects the relative continuity of thematic linkages across time. The narrow range of stability values in the table indicates that the strongest thematic flows displayed broadly similar levels of continuity. Therefore, differences among these flows should be interpreted mainly through their Inc_Weighted values rather than through small variations in stability.

As shown in Table 2, the theme *child abuse* demonstrates strong continuity from the 2000–2012 period to the 2013–2019 period (Inc_Weighted = 0.725), representing the highest carryover value in the dataset. Similarly, *child maltreatment* shows substantial persistence from 2013 to 2019 into the 2020–2025 period (Inc_Weighted = 0.696). The results further indicate cross thematic linkages across periods. In particular, *child welfare* in 2013–2019 is connected with *child maltreatment* in 2020–2025 (Inc_Weighted = 0.574). In addition, *child abuse* in 2013–2019 is linked with *child protection* in the subsequent period (Inc_Weighted = 0.558), indicating continuity across related thematic domains. A further transition was observed between child maltreatment in 2000–2012 and intimate partner violence in 2013–2019 (Inc_Weighted = 0.520). This value indicates a cross-period thematic linkage in the thematic evolution analysis rather than showing intimate partner violence as a dominant standalone cluster in the thematic map. Across the identified flows, several core themes, particularly *child abuse* and *child maltreatment*, appear repeatedly in high strength transitions. These values describe measurable patterns of thematic continuity and reconfiguration across successive periods in the literature. The stability values were closely clustered across the top flows, suggesting that these major thematic transitions had comparable levels of temporal continuity. For this reason, the relative strength of the flows was interpreted primarily according to the Inc_Weighted values.

## Discussion

This study examined the development of the child neglect and abuse literature within the social work context between 2000 and 2025 by integrating quantitative growth indicators with patterns of thematic transformation and continuity. The findings indicate that the field has not only expanded in publication volume but has also moved toward a more structured and system-oriented knowledge base organized around several core themes. This pattern is consistent with prior scholarship that has questioned narrow, individually focused and family-pathology explanations of child maltreatment and has increasingly emphasized child welfare systems, institutional responses, poverty, material hardship, and social policy frameworks (7, 9–11). The observed increase in annual publication output and the calculated growth indicators suggest sustained scholarly attention to child neglect and abuse over the past 25 years. The temporal publication curve derived from the bibliometric dataset shows that publication growth became more visible from approximately 2008 onward and gained further momentum after 2016. This increase does not appear to represent only a numerical expansion of the literature. Rather, it points to a period in which child maltreatment became more firmly positioned as a public health priority, a child welfare issue, and a social work concern. The growing visibility of prevention-oriented and system-level research further aligns with literature emphasizing the substantial health, social, and economic burden of child maltreatment and the need to situate prevention within broader health, welfare, and policy agendas (3, 27, 28).

The analysis of highly cited publications reveals a markedly skewed citation distribution, with a relatively small number of studies accounting for a substantial share of total citations. Co-citation concentration around a limited set of influential works indicates the presence of a well-defined intellectual core within the field. Bibliographic coupling patterns further suggest that this core is not limited to earlier foundational works but is also carried forward through more recent author communities that draw on overlapping bodies of literature. Many of these highly cited studies address issues with clear policy and practice relevance, including parenting stress and maltreatment risk, mental health service needs, maltreatment risk factors, disability-related vulnerability, economic burden, intervention effectiveness, neighborhood conditions, and child welfare system involvement (27, 29–35). This pattern suggests that research generating evidence relevant to prevention, intervention, service planning, and policy development tends to achieve greater scientific visibility. This emphasis on prevention- and intervention-oriented evidence is also reflected in MacMillan et al. (2009), who reviewed strategies to prevent child maltreatment and associated impairment and stressed the need for rigorously evaluated interventions using maltreatment and health-related outcomes (36). From a social work perspective, the prominence of these themes underscores the field's applied, system-sensitive, and intervention-oriented knowledge base, particularly where research connects maltreatment outcomes with child welfare inequalities, service responses, and institutional decision-making (8, 37).

The concentration of collaboration in a small group of high-income Anglophone countries should be interpreted not only as a pattern of scientific productivity but also as a potential indication of unequal visibility in global child welfare knowledge production. In other words, the collaboration map shows where research is most visible in the indexed literature, but not necessarily where child protection problems are most urgent or complex. The centrality of the United States, the United Kingdom, Canada, and Australia may partly reflect stronger research infrastructures, the longer institutional development of statutory child protection systems, and the closer integration of child welfare research with policy, legal frameworks, administrative processes, and professional infrastructures in these settings (9, 38). Indeed, previous bibliometric research on child maltreatment has consistently identified the United States as the most productive country in the field (13). This pattern may also point to a structural imbalance in the bibliometric record, as the coverage of Web of Science and Scopus is known to be geographically uneven, with Europe and North America generally overrepresented and some regions in Asia, Africa, and Latin America less visible in these databases (39). From a social work perspective, this imbalance is important because child protection systems are not organized around a single universal model; rather, they are shaped by welfare regimes, legal definitions of maltreatment, criteria for state intervention, administrative referral processes, reporting systems, and placement arrangements (38). These system-level differences intersect with poverty structures and social inequalities, indicating that child maltreatment and child protection should be examined across diverse welfare, legal, and socio-economic contexts rather than generalized from a narrow set of dominant Anglophone systems (7, 8).

Keyword co-occurrence and thematic evolution analyses provide a clearer picture of how the field has been conceptually organized over time. The literature appears to revolve around a set of stable core themes, particularly child abuse, child maltreatment, child welfare, and child protection. Among these terms, child maltreatment has gained increasing prominence in the keyword network, suggesting that it has gradually come to function as a broader integrative concept linking different forms of harm, unmet needs, developmental consequences, and their relevance for child welfare systems. This interpretation is consistent with scholarship that treats child maltreatment not as a single incident or narrowly bounded category, but as a multidimensional construct involving abuse, neglect, definitional complexity, and long-term consequences for children and families (11, 40, 41).

The thematic evolution results also suggest that the field has not developed through a simple linear accumulation of topics. Rather, it shows a combination of continuity and periodic reconfiguration. The continued presence of child abuse from the earliest period points to an important historical continuity, rooted in the recognition of physical abuse as a distinct medical and social problem in the early child maltreatment literature (42). At the same time, the growing visibility of child welfare and child protection in later periods indicates that the field has increasingly moved toward questions of systems, policy, professional practice, and institutional response. The weighted thematic flow analysis (Inc_Weighted) supports this reading by showing strong cross-period linkages among these core themes. This pattern is also in line with scholarship showing that contemporary child protection practice is shaped not only by the characteristics of individual cases, but also by organisational cultures, legal thresholds, managerial hierarchies, risk-management logics, service resources, workload pressures, and wider social inequalities (9, 41, 43).

Importantly, the divergence between rising publication counts and the post-2020 decline in annual citation totals should be interpreted cautiously. Because bibliometric citation accumulation is inherently time-lagged, recently published studies have had less opportunity to accrue citations, which likely contributes to the observed downturn in the most recent years. This temporal citation effect is widely documented in bibliometric research and should not be interpreted as a decline in scholarly influence. These findings should be interpreted within the methodological boundaries of the present bibliometric design, including database coverage, language restrictions, and threshold parameters applied during network construction. Within these parameters, the results provide a systematic account of how the field has expanded, consolidated, and reoriented its thematic priorities over the past quarter century.

## Limitations

The findings of this study should be interpreted in light of several methodological constraints inherent to bibliometric research. The dataset was restricted to publications indexed in the Web of Science Core Collection and Scopus. Although these databases provide broad coverage of peer reviewed literature in the social sciences, they may not fully capture regional journals, books, and policy reports. This limitation is particularly relevant for social work research, as coverage differences between Web of Science and Scopus may affect the visibility of regional child welfare and child protection journals, including Nordic and continental European outlets. This may have contributed to the observed concentration of publications and collaborations in Anglophone and high-income countries. Therefore, the geographic findings should be interpreted as patterns within the indexed literature rather than as a complete representation of global social work knowledge production. As a result, certain strands of practice oriented or locally grounded social work scholarship may be underrepresented in the present mapping. The analysis was limited to English language publications in order to maintain consistency in keyword processing and cross database comparability. While this decision strengthens standardization, it also reduces the visibility of research produced in diverse linguistic and regional contexts. Given the strong influence of national welfare regimes on child protection practice, some context specific knowledge may remain outside the analytical frame.

Citation-based indicators should also be interpreted with caution. Citation counts are useful for identifying patterns of scholarly visibility, yet they do not necessarily reflect practical impact, policy uptake, or field level implementation. Conceptual and epidemiological studies therefore tend to appear more prominent than applied intervention research that directly informs frontline social work practice. The thematic analyses rely on author provided keywords and thesaurus-based harmonization. Although these procedures reduce terminological fragmentation, keyword selection ultimately reflects authors' labeling preferences and may not fully capture the conceptual depth of individual studies. The thematic structures reported here should therefore be interpreted as indicators of dominant research tendencies rather than exhaustive representations of the field. Finally, recent publications have had less time to accumulate citations. This time lag effect may influence the relative visibility of the most recent research developments in the citation-based findings.

## Conclusion

This study examined the development of the child neglect and abuse literature within the social work context between 2000 and 2025 using a comprehensive bibliometric framework based on the combined Web of Science and Scopus dataset. The findings indicate that the field has expanded steadily in quantitative terms while also undergoing a process of thematic consolidation and structural reorientation. The sustained rise in publication output confirms that child neglect and abuse has become a firmly established domain within social work scholarship. More importantly, the bibliometric evidence demonstrates that the field is not expanding randomly but is reorganizing around a more system aware and prevention-oriented knowledge structure. While child abuse and child maltreatment continue to form the conceptual backbone of the literature, child welfare and child protection themes have moved toward the center of the research landscape. This shift reflects the growing alignment of social work research with system level intervention, service coordination, and policy responsive practice.

The collaboration networks further reveal that knowledge production in this area remains concentrated within a limited group of countries and research institutions. From a social work perspective, this pattern signals the importance of strengthening cross national collaboration and increasing the visibility of diverse child protection contexts, particularly those operating under different welfare regimes. By mapping growth dynamics, intellectual structure, and thematic evolution within a single analytical framework, this study provides one of the most comprehensive field level portraits of child neglect and abuse research in the social work tradition. The findings offer an empirical baseline for monitoring how the field continues to evolve and where conceptual and geographical gaps remain.

Future research would benefit from broader database integration, multilingual expansion, and closer linkage between bibliometric patterns and practice-oriented evidence. Comparative and cross-national designs are likely to be especially valuable for advancing context sensitive knowledge in child protection. For social work practice and policy, the observed movement toward welfare and protection system themes underscores the need to prioritize preventive services, interagency coordination, and evidence informed decision processes. Strengthening the bridge between research production and child protection practice remains a central task for the field.

## Data Availability

The original contributions presented in the study are included in the article/Supplementary Material, further inquiries can be directed to the corresponding author.
